# Longitudinal study of body mass index, dyslipidemia, hyperglycemia, and hypertension in 60,000 men and women in Sweden and Austria

**DOI:** 10.1371/journal.pone.0197830

**Published:** 2018-06-13

**Authors:** Mieke Van Hemelrijck, Hanno Ulmer, Gabriele Nagel, Raphael Simon Peter, Josef Fritz, Robin Myte, Bethany van Guelpen, Bernhard Föger, Hans Concin, Christel Häggström, Pär Stattin, Tanja Stocks

**Affiliations:** 1 King’s College London, Division of Cancer Studies, Translational Oncology & Urology Research, London, United Kingdom; 2 Medical University Innsbruck, Department of Medical Statistics, Informatics, and Health Economics, Innsbruck, Austria; 3 Ulm University, Institute of Epidemiology and Medical Biometry, Ulm, Germany; 4 Agency for Preventive and Social Medicine, Bregenz, Austria; 5 Umeå University, Department of Radiation Sciences, Umeå, Sweden; 6 Umeå University, Department of Biobank Research, Umeå, Sweden; 7 Uppsala University, Department of Surgical Sciences, Uppsala, Sweden; 8 Umeå University, Department of Public Health and Clinical Medicine, Nutritional Research, Umeå, Sweden; 9 Lund University, Department of Clinical Sciences Malmö, Malmö, Sweden; Shanghai Diabetes Institute, CHINA

## Abstract

**Background:**

Obesity is suggested to underlie development of other metabolic aberrations, but longitudinal relationships between metabolic factors at various ages has not been studied in detail.

**Methods:**

Data from 27,379 men and 32,275 women with in total 122,940 health examinations in the Västerbotten Intervention Project, Sweden and the Vorarlberg Health Monitoring and Prevention Programme, Austria were used to investigate body mass index (BMI), mid-blood pressure, and fasting levels of glucose, triglycerides, and total cholesterol at baseline in relation to 10-year changes of these factors and weight. We included paired examinations performed 10±2 years apart and used them for longitudinal analysis with linear regression of changes between the ages 30 and 40, 40 and 50, or 50 and 60 years.

**Results:**

Higher levels of BMI were associated with increases in glucose and mid-blood pressure as well as triglycerides levels, and, to a lesser extent, decreases in cholesterol levels. For instance, per 5 kg/m^2^ higher BMI at age 40, glucose at age 50 increased by 0.24 mmol/l (95%CI: 0.22–0.26) and mid-blood pressure increased by 1.54 mm Hg (95%CI: 1.35–1.74). The strongest association observed was between BMI at age 30 and mid-blood pressure, which was 2.12 mm Hg (95% CI: 1.79–2.45) increase over ten years per 5 kg/m^2^ higher BMI level. This association was observed at an age when blood pressure levels on average remained stable. Other associations than those with BMI at baseline were much weaker. However, triglyceride levels were associated with future glucose changes among individuals with elevated BMI, particularly in the two older age groups.

**Conclusion:**

BMI was most indicative of long-term changes in metabolic factors, and the strongest impact was observed for increases in blood pressure between 30 and 40 years of age. Our study supports that lifestyle interventions preventing metabolic aberrations should focus on avoiding weight increases.

## Introduction

Overweight and obesity, hypertension, high blood glucose, high triglycerides, and reduced high-density lipoprotein cholesterol, have all been indicated as strong risk factors for cardiovascular disease, cancer and diabetes [1]. Given that most of these “metabolic factors” are preventable and afflicted subjects have a strongly increased future risk of cardiovascular disease, it is of interest to study these metabolic factors and their correlations longitudinally.

Several studies suggest that obesity is key in development of other metabolic factors [2, 3]. A longitudinal survey in Mauritius (5 and 11 years) and Australia (5 years) showed that high waist circumference predicted deterioration in other metabolic factors [4]. The Dutch Doetinchem Cohort study showed an association of weight change with the number of metabolic aberration that was strongest in the younger people [5]. A Danish study based on data from the national service found no evidence for the hypothesis that rapid increases in body mass index (BMI) in childhood or early-onset obesity were associated with ‘metabolically healthy obesity’ [6]. Another study based on Study of Health in Pomerania used a network-based approach to study changes in metabolic factors over a period of five years. They identified that hypertension and central obesity were the most common metabolic factors, but low HDL cholesterol was found to be the most common new onset metabolic factor [7].

To our knowledge little information is available on age and sex-specific trajectories for these metabolic factors, especially in young adulthood when prevention may be of particular importance to prevent or delay development of metabolic diseases. These trajectories can help elucidate how baseline metabolic factors in adults predict their longitudinal changes; a question that urgently needs answers to guide ongoing development of lifestyle interventions and to inform policies aimed at reducing the global burden of diseases related to metabolic aberrations. Here, we aimed to assess how metabolic factors at baseline predict their longitudinal changes and interactions, specific for ages 30 to 40, 40 to 50, and 50 to 60 years in two large European cohorts of nearly 60,000 men and women.

## Materials and methods

### Participants and health examinations

We combined data from the Västerbotten Intervention Project (VIP) [8, 9] with the Vorarlberg Health Monitoring and Prevention Programme (VHM&PP) [10], as they both comprise longitudinal data on metabolic factors. The VIP in northern Sweden and the VHM&PP in the most western province of Austria have previously been described in detail [8–10]. VIP started in 1985 and is an ongoing health survey of inhabitants of Västerbotten county (N~270,000 in 2017) for the prevention of cardiometabolic disease. All residents in the county are invited to a health examination at the age of 40, 50 and 60 years, previously also at the age of 30 years. Throughout the years, between 48 and 67% of the eligible population attended the examination [11]. Participants receive lifestyle counselling and, if needed, pharmaceutical treatments based on the results of the examination. Since 1992, participants have been instructed to fast over night before the examination. The examination is performed according to a standardized protocol by a trained nurse and includes measurements of height, weight, blood pressure, and fresh plasma measures of glucose, total cholesterol, and triglycerides. Height and weight are measured in light clothing and without shoes, and blood pressure is measured after five minutes of rest using a mercury sphygmomanometer. Before Sept 1, 2009, blood pressure was measured in supine position and was thereafter measured in a sitting position. Glucose levels are measured by Reflotron bench-top analyzers (Boerhinger Mannheim, Germany), which before Sept 1, 2009 was also used for the measurement of triglycerides and total cholesterol. After that date, triglycerides and cholesterol were measured at the clinical chemistry department at the nearest hospital using standard enzymatic methods. For research purposes, algorithms have been developed to transform levels between new and old measurement methods of blood pressure and plasma lipids, which we used in the present study. These algorithms were based on individuals with measurements using both old and new measurements methods (n = 838 for triglycerides, 1197 for cholesterol, and 648 for blood pressure). Triglyceride and cholesterol levels measured from Sept 1, 2009 onwards were converted to old measurement levels using the formulas: 0.177+(0.932 × triglyceride level) and 0.170+(0.939 × cholesterol level). Formulas for blood pressure were age- and sex-specific (S1 Table). In addition to clinical measurements, participants were also asked to fill in a questionnaire including questions on lifestyle and medication. The present study includes questionnaire data on smoking status (never-, ex-, or current-smoker), prevalent diabetes (“Do you have diabetes?”) and antihypertensive use (“Have you used antihypertensive medication during the past 14 days?”).

The VHM&PP is a health survey of inhabitants of the Vorarlberg province (N~390,000 in 2017) conducted between 1985 and 2005 with the aim to prevent chronic diseases, cardiovascular diseases in particular. Inhabitants of the province aged 19 years or more were invited to annual health examinations, and more than 60% of the eligible population has participated in the programme [12]. Health examinations were performed according to a standardized protocol by a trained general practitioner. Participants received lifestyle advice based on the results of the examination. Since 1988, participants were instructed to fast overnight before the examination. Measurements included height, weight, blood pressure, and plasma measures of glucose, total cholesterol, and triglycerides. Height and weight were measured in light clothing and without shoes, and blood pressure was measured in a sitting position after five minutes of rest using a mercury sphygmomanometer. Glucose, cholesterol and triglycerides were measured by enzymatic methods at two laboratories in the region. Smoking status (never-, ex-, or current-smoker) was asked by the examining physician who recorded the response on an examination sheet.

Participants in the two cohorts have been linked to their respective cancer registry, which was used for the participant selection of the present study. Data of cancer diagnoses were available from 1958 (the VIP) or 1985 (the VHM&PP), until Dec 31, 2014. The study received approval by the ethical committees of Umeå University, Sweden (2012-354-31M) and of the Vorarlberg region, Austria (2006-6/2).

### Selection criteria

Out of 296,403 individuals with 882,109 health examinations, we excluded: 40 examinations with non-matching data in cohorts compared to registries (e.g. gender), 20,316 examinations with a date of diagnosis of an invasive cancer before or at the date of health examination, 101,935 examinations performed in a non-fasting state (<8 h) or with fasting status missing, 28 examinations with no data on one of the metabolic factors evaluated in this study (i.e. BMI, blood pressure, glucose, triglycerides or total cholesterol), and 120,911 individuals with only one health examination after which 153,585 individuals (34,953 in the VIP and 118,632 in the VHM&PP) with 638,879 health examinations (73,509 in the VIP and 565,370 in the VHM&PP) were eligible for analysis in this study.

Due to the nature of health examinations at 30, 40, 50, and 60 years of age in the VIP, we decided to mimic this age categorization in the VHM&PP rather than comparing results from various ages and durations in the longitudinal analyses. Thus, we only included examinations that had been performed at 30, 40, 50 or 60 years of age ±2 years, and if several examinations were available for an age interval, we selected the one closest to the center of the interval. Of these examinations, we included paired examinations that had been carried out 10±2 years apart and could therefore be used for the longitudinal analysis between 30 and 40 years, 40 and 50 years, or 50 and 60 years of age. In total, 59,654 individuals—33,802 from the VIP and 25,852 from the VHM&PP—were included, of whom 56,022 had data available for the analysis of changes of metabolic factors within one age-period, and the remaining 3,632 individuals had data available for two age-periods. All participants thus had at least 10 years of follow-up and all serum-based metabolic factors were measured in fasting samples.

### Statistical analysis

All analyses were calculated using age-, sex- and cohort-specific Z-transformed values of metabolic factors with a mean value of zero and a standard deviation (SD) of one applying the formula (metabolic level-mean)/SD on log-transformed values, within an individual’s age (30, 40, 50 or 60 years), sex and cohort. Blood pressure was studied as mid-blood pressure, i.e. the mean of systolic plus diastolic blood pressure, due to its relevance in cardiovascular mortality [13]. Among 30-year olds in our study, amongst whom little use and therefore little influence of medication on blood pressure values can be expected, mid-blood pressure was highly correlated with systolic blood pressure (r = 0.85), diastolic blood pressure (r = 0.92) and mean arterial pressure (r = 0.98). We first calculated partial correlation coefficients between metabolic factors measured at 30, 40, and 50 years, respectively, adjusted for smoking status (never-/ex-/current-smoker or missing [<1%]). Participants presenting with extreme values (specifically, a Z-transformed value >3 or <-3) were excluded for analysis of the respective time-point. Correlation coefficients from these analyses are reported in S1 Fig and were, together with results from linear regression analyses, used to determine adjustment factors in the final linear regression models.

Linear regression was used to investigate the association between each metabolic factor at 30, 40 and 50 (±2) years of age, in relation to the annual change (log- and Z-transformed) of another metabolic factor measured at the time of exposure measurement and ten years later (±2 years). Due to the use of Z-transformed exposure and outcome variables, effect sizes from linear regression analyses were directly comparable and were interpreted as the annual unit change of a metabolic factor expressed in SDs, by each SD increment of another metabolic factor measured at baseline. Analyses of BMI as exposure were additionally performed for per 5 kg/m^2^ increment of BMI in relation to the 10-year SI unit change (10 × annual change) of other metabolic factors. All analyses were adjusted for baseline smoking status and baseline level of the outcome metabolic factor due to their potential of being confounders owing to their relationships with metabolic factors and their long-term changes. As BMI was modestly correlated with each of the other metabolic factors (r≤0.41) and was the main factor influencing changes of other factors, we adjusted all linear regression analyses for baseline BMI as a potential confounder, with the exception of the analyses in which weight change was the outcome, which instead was adjusted for baseline weight (r>0.80 with BMI). Additionally, based on a modest correlation between cholesterol and triglycerides (r≤0.41), and a potential mutual influence of these factors on each other in some of the linear regression analyses, the final linear regression analyses of cholesterol and triglycerides were mutually adjusted for the baseline level of the other factor. Each analysis excluded individuals with values more extreme than ±3 SDs of the exposure, outcome, or baseline level of the outcome metabolic factor (maximum 4% of individuals) since a high proportion of these measured levels are likely to be erroneous or influenced by medication (change in metabolic factor), and because outliers in a linear model may disproportionally influence the results. Whenever blood pressure was the exposure or blood pressure change was the outcome, we performed sensitivity analyses among VIP participants with additional exclusions of individuals on antihypertensive drugs at baseline or at baseline and/or follow-up, respectively. In the same manner, sensitivity analyses of glucose excluded participants with diabetes. We investigated interactions between baseline BMI and the other metabolic factors by evaluating the Wald test for a product term of BMI and a metabolic factor. Stata version 13.1 was used for statistical analyses.

## Results

A total of 5,208 men and 6,353 women were aged 30, 15,801 men and 18,844 women were aged 40, 22,853 men and 26,718 women aged 50, and 12,508 men and 14,655 women were aged 60, Table 1. The proportion of abnormal metabolic factors increased by age. For instance, the proportion of obese men (and women) increased from 9 to 12% between the age of 30 and 40, to 15% at age 50, and 18% at age 60 in the VIP cohort.

**Table 1:** Characteristics of the 59,654 individuals with 122,940 health examinations in the Västerbotten Intervention Project and the Vorarlberg Health Monitoring and Prevention Programme by age of measurement [31–34].

Age	30 years		40 years		50 years		60 years	
	Men	Women	Men	Women	Men	Women	Men	Women
Number of examinations								
VIP	1671	1848	8451	9575	15,047	16,689	8501	9388
VHM&PP	3537	4505	7350	9269	7806	10,029	4007	5267
Year of examination, median (range)								
VIP	1994 (1992–2006)		1999 (1992–2014)		2003 (1992–2014)		2009 (2001–2014)	
VHM&PP	1992 (1988–1997)		1996 (1988–2005)		1996 (1988–2005)		2001 (1996–2005)	
Smokers^a^, %								
VIP	9	17	12	17	14	17	13	12
VHM&PP	35	32	31	27	24	20	18	14
BMI (kg/m^2^)^a^, median (pc 25–75)								
VIP	24.7 (22.6–26.9)	23.0 (21.3–25.5)	25.6 (23.7–27.8)	23.8 (21.9–26.6)	26.2 (24.3–28.4)	24.8 (22.7–27.8)	26.6 (24.6–29.0)	25.6 (23.3–28.7)
VHM&PP	24.2 (22.4–26.2)	21.6 (20.0–24.0)	25.2 (23.4–27.5)	23.0 (21.0–25.9)	26.0 (24.1–28.3)	24.7 (22.3–27.9)	26.6 (24.6–29.1)	25.8 (23.3–29.1)
Obese (BMI ≥30 kg/m^2^)^a^^,^^b^, %								
VIP	9	8	12	11	15	15	18	19
VHM&PP	5	5	10	9	14	15	18	21
Systolic blood pressure (mm Hg)^a^, median (pc 25–75)								
VIP	120 (115–130)	115 (108–120)	120 (115–130)	115 (108–122)	125 (118–136)	120 (112–133)	132 (122–142)	129 (120–140)
VHM&PP	125 (120–132)	115 (110–125)	125 (120–135)	120 (110–130)	130 (120–140)	130 (120–140)	140 (125–150)	140 (125–150)
Diastolic blood pressure (mm Hg)^a^, median (pc 25–75)								
VIP	75 (70–80)	70 (65–77)	76 (70–82)	71 (65–80)	80 (75–87)	77 (70–84)	79 (73–85)	82 (76–88)
VHM&PP	80 (75–85)	75 (70–80)	80 (75–85)	80 (70–80)	80 (80–90)	80 (75–90)	80 (80–90)	80 (80–90)
Mid-blood pressure (mm Hg)^a^, median (pc 25–75)								
VIP	99 (93–105)	93 (88–98)	100 (93–105)	93 (88–100)	103 (96–111)	100 (92–108)	107 (100–115)	104 (96–111)
VHM&PP	103 (95–109)	95 (90–103)	103 (98–110)	100 (90–105)	105 (100–115)	105 (98–114)	110 (103–120)	110 (101–118)
Hypertension (≥140/90 mm Hg, or in the VIP, self-reported hypertension or antihypertensive drug use)^a^^,^^b^, %								
VIP	16	16	21	19	38	34	58	52
VHM&PP	27	11	31	19	45	39	57	55
Glucose (mmol/l)^a^, median (pc 25–75)								
VIP	5.1 (4.8–5.5)	5.1 (4.7–5.4)	5.4 (5.0–5.8)	5.3 (5.0–5.7)	5.5 (5.0–5.9)	5.4 (5.0–5.8)	5.6 (5.1–6.1)	5.4 (5.0–5.9)
VHM&PP	4.5 (4.1–4.9)	4.4 (4.0–4.9)	4.8 (4.4–5.3)	4.7 (4.3–5.1)	5.0 (4.5–5.5)	4.8 (4.4–5.3)	5.3 (4.8–5.8)	5.1 (4.7–5.6)
Diabetic fasting glucose (≥7.0 mmol/l) or in the VIP, self-reported diabetes^a^^,^^b^, %								
VIP	0	1	1	1	3	2	9	5
VHM&PP	1	0	2	1	4	2	7	4
Total cholesterol (mmol/l)^a^, median (pc 25–75)								
VIP	5.0 (4.4–5.7)	4.8 (4.2–5.4)	5.3 (4.7–6.1)	5.0 (4.4–5.6)	5.6 (4.9–6.3)	5.4 (4.9–6.1)	5.4 (4.7–6.1)	5.8 (5.1–6.4)
VHM&PP	5.2 (4.6–5.9)	4.9 (4.4–5.6)	5.6 (4.9–6.3)	5.2 (4.6–5.8)	5.8 (5.1–6.6)	5.7 (5.1–6.4)	5.8 (5.1–6.5)	6.1 (5.5–6.8)
High cholesterol (≥6.2 mmol/l)^a^^,^^b^, %								
VIP	15	8	21	10	28	22	21	32
VHM&PP	18	11	30	15	37	31	34	46
Triglycerides (mmol/l)^a^, median (pc 25–75)								
VIP	1.1 (0.8–1.6)	0.9 (0.8–1.3)	1.2 (0.9–1.8)	0.9 (0.8–1.2)	1.3 (1.0–1.9)	1.1 (0.8–1.4)	1.3 (1.0–1.8)	1.2 (0.9–1.6)
VHM&PP	1.2 (0.8–1.8)	1.0 (0.7–1.3)	1.4 (0.9–2.1)	0.9 (0.7–1.3)	1.4 (1.0–2.2)	1.1 (0.8–1.5)	1.4 (1.0–2.1)	1.2 (0.9–1.7)
High triglycerides (≥2.3 mmol/l)^a^^,^^b^, %								
VIP	10	3	12	3	15	6	13	8
VHM&PP	14	4	21	4	22	8	20	10

VIP, Västerbotten Intervention Project; VHM&PP, Vorarlberg Health Monitoring and Prevention Programme; BMI, body mass index; pc, percentile.

^a^The number (%) of missing values for each variable out of 122,940 health examinations was for: marital status, 888 (1); smoking, 51 (0); BMI, 147 (0); systolic blood pressure, 387 (0); diastolic blood pressure, 437 (0); glucose, 209 (0); cholesterol, 309 (0); and triglycerides, 3569 (3).

^b^Sources for categories: BMI, World Health Organization, 2008; blood pressure, Whithworth, 2003; glucose, World Health Organization, 1999; and cholesterol and triglycerides, Jellinger et al., 2017.

Table 2 shows the median 10-year change of each metabolic factor by age, sex, and cohort. Overall, metabolic levels increased in all age groups apart from mid-blood pressure, which tended to increase only from age 40. The median change in weight decreased by age (e.g. men in the VHM&PP aged 30–40 vs aged 50–60: 3.61 vs 1.93 kg). No clear differences by age were observed for the other metabolic factors.

**Table 2:** Median (percentile 25–75) 10-year^a^ changes of metabolic factors by age, sex and cohort.

Metabolic factor	Cohort	30–40 years^b^^,^^c^		40–50 years^b^^,^^d^		50–60 years^b^^,^^e^	
		Men	Women	Men	Women	Men	Women
Weight, kg	VIP	5.09 (1.04; 9.38)	3.96 (0.00; 7.90)	3.11 (0.00; 7.00)	3.00 (0.00; 6.73)	1.99 (-1.03; 5.03)	1.99 (-1.04; 5.11)
	VHM&PP	3.61 (0.00; 7.42)	3.32 (0.00; 7.00)	2.84 (0.00; 6.09)	3.31 (0.00; 7.06)	1.93 (-1.00; 4.91)	2.43 (-0.50; 5.84)
Mid-blood pressure, mm Hg	VIP	0.00 (-5.49; 7.02)	0.00 (-5.44; 7.01)	3.98 (-2.64; 10.8)	4.84 (-2.13; 11.4)	3.57 (-4.14; 11.6)	3.01 (-4.96; 10.5)
	VHM&PP	0.00 (-7.19; 8.52)	1.31 (-5.92; 9.16)	2.49 (-5.34; 10.6)	4.44 (-4.55; 12.5)	2.63 (-5.77; 11.6)	2.98 (-5.34; 12.4)
Glucose, mmol/l	VIP	0.21 (-0.21; 0.71)	0.20 (-0.29; 0.67)	0.00 (-0.50; 0.52)	0.00 (-0.41; 0.51)	0.10 (-0.49; 0.60)	0.00 (-0.42; 0.57)
	VHM&PP	0.51 (-0.10; 1.14)	0.37 (-0.17; 0.96)	0.55 (-0.10; 1.23)	0.43 (-0.15; 1.02)	0.56 (-0.11; 1.22)	0.49 (-0.12; 1.09)
Cholesterol, mmol/l	VIP	0.13 (-0.48; 0.68)	0.00 (-0.50; 0.53)	0.12 (-0.51; 0.71)	0.34 (-0.21; 0.86)	-0.22 (-0.98; 0.40)	0.28 (-0.39; 0.87)
	VHM&PP	0.28 (-0.28; 0.84)	0.20 (-0.34; 0.71)	0.07 (-0.56; 0.66)	0.43 (-0.12; 0.98)	-0.11 (-0.75; 0.49)	0.35 (-0.31; 0.93)
Triglycerides, mmol/l	VIP	0.11 (-0.19; 0.52)	0.00 (-0.18; 0.23)	0.12 (-0.20; 0.52)	0.07 (-0.10; 0.35)	0.02 (-0.32; 0.38)	0.10 (-0.14; 0.40)
	VHM&PP	0.06 (-0.32; 0.53)	-0.09 (-0.37; 0.18)	-0.07 (-0.53; 0.37)	0.05 (-0.21; 0.36)	-0.10 (-0.59; 0.33)	0.08 (-0.21; 0.41)

VIP, Västerbotten Intervention Project; VHM&PP, Vorarlberg Health Monitoring and Prevention Programme.

^a^Measurements were taken 8–12 years apart, but changes in metabolic factor levels were projected to the time period of ten years.

^b^The number of individuals in each analysis differed due to various completeness of metabolic factors, with the most missing values for triglycerides in the VIP.

^c^Number of individuals in each analysis: VIP-Men = 1224–1665, VIP-Women = 1710–1838, VHM&PP-Men = 3528–3537, VHM&PP-Women = 4491–4505.

^d^Number of individuals in each analysis: VIP-Men = 6318–7443, VIP-Women = 8162–8478, VHM&PP-Men = 3811–3818, VHM&PP-Women = 4757–4784.

^e^Number of individuals in each analysis: VIP-Men = 7474–8465, VIP-Women = 8991–9356, VHM&PP-Men = 3994–4007, VHM&PP-Women = 5251–5267.

**Fig 1:**
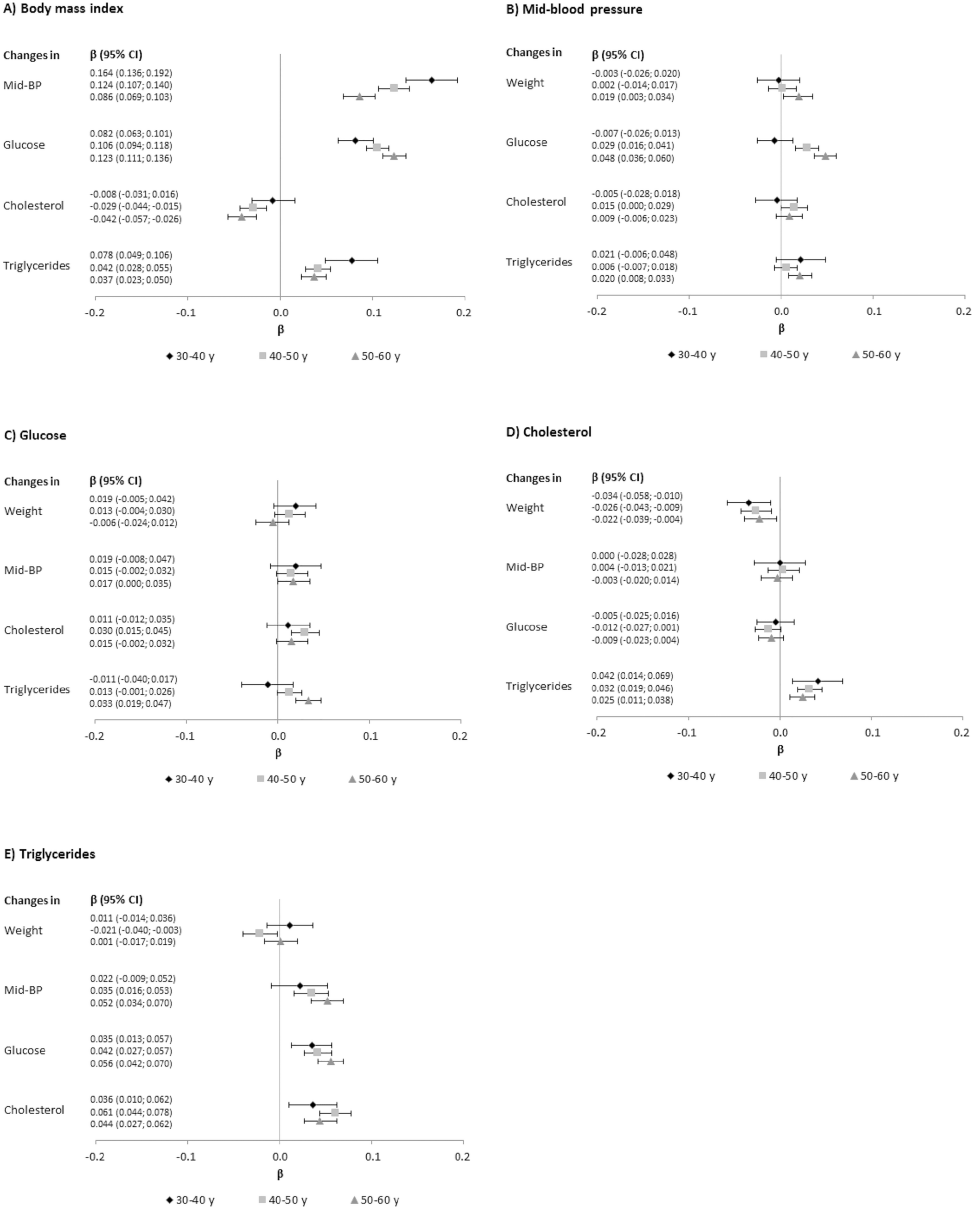
Beta (β) and 95% confidence intervals (CI) from linear regression with baseline A) body mass index, B) mid-blood pressure, C) glucose, D) total cholesterol, and E) triglycerides as exposure, and change in a metabolic factor as outcome, by age (baseline-end of follow-up). Analyses were adjusted for baseline smoking status and baseline level of the outcome metabolic factor and body mass index (except in A). Analyses of cholesterol and triglycerides as exposures were additionally mutually adjusted for baseline level of the counterpart factor. All metabolic factors, and annual change of the outcome metabolic factor, were log-transformed and entered into the model on their Z transformed scale, standardized by sex and cohort. Each analysis excluded individuals with values more extreme than ±3 standard deviations of the exposure, outcome, or baseline level of the outcome metabolic factor. The number of individuals in each analysis differed depending on completeness of variables and on exclusions and was: 30–40 years, 5253–8388; 40–50 years, 12 442–17 137; 50–60 years, 13 345–16 694. Abbreviation; BP, blood pressure; CI, confidence interval; y, years.

Fig 1A–1E show how each baseline metabolic factor predicted 10-year changes in the other metabolic factors studied, with numbers denoting the per SD increment of an outcome factor by each SD higher level of a baseline metabolic factor. Cohort- and sex-specific estimates are shown in S2A–S2E Fig. The strongest associations were seen for baseline BMI, in particular for mid-blood pressure and glucose (Fig 1A). For example, increase per SD in BMI at age 40, mid-blood pressure at ages 40 and 50 increased 0.12 (95%CI: 0.11–0.14) SDs and glucose increased 0.11 SDs (95%CI: 0.09–0.12). In the same ages, each 5 kg/m^2^ higher BMI was associated with an absolute increase in 10-year mid-blood pressure of 1.54 mm Hg (95%CI: 1.35–1.74) (1.12 to 2.26 mm Hg in cohort- and sex-specific estimates) and a 10-year glucose increment of 0.24 mmol/l (95%CI: 0.22–0.26) (Table 3) (0.17 to 0.36 mmol/l in cohort- and sex-specific estimates). The strength of association between BMI and changes in mid-blood pressure and glucose, respectively, decreased with increasing age for mid-blood pressure and increased with increasing age for glucose. Baseline BMI was also positively associated with changes in triglycerides. In contrast, there was a weak inverse association with cholesterol levels.

**Table 3:** Results in Fig 1A when using body mass index per 5 kg/m^2^ increment as exposure and absolute unit changes (95% confidence intervals) of metabolic factors over ten years as outcomes^a^.

Outcome factor, change in	30–40 years	40–50 years	50–60 years
Mid-blood pressure, mm Hg	2.12 (1.79; 2.45)	1.54 (1.35; 1.74)	1.23 (1.03; 1.44)
Glucose, mmol/l	0.14 (0.11; 0.16)	0.24 (0.22; 0.26)	0.26 (0.24; 0.29)
Cholesterol, mmol/l	0.00 (-0.02; 0.03)	-0.02 (-0.04; -0.01)	-0.04 (-0.05; -0.02)
Triglycerides, mmol/l	0.06 (0.03; 0.10)	0.04 (0.02; 0.06)	0.03 (0.01; 0.04)

^a^Exclusions and adjustments were the same as in the analysis of Fig 1A, with the addition of adjustment for sex and cohort.

Metabolic factors other than BMI showed much weaker associations with changes in other metabolic factors (Fig 1B–1E). The most consistent associations were shown for baseline triglycerides, which were positively associated with changes in mid-blood pressure, glucose, and cholesterol (Fig 1E). Sensitivity analyses in the VIP excluding participants on antihypertensive drugs in analyses of mid-blood pressure, and excluding diabetic participants in analyses of glucose, had a minor impact on the results from longitudinal analyses (S3 Fig).

**Fig 2:**
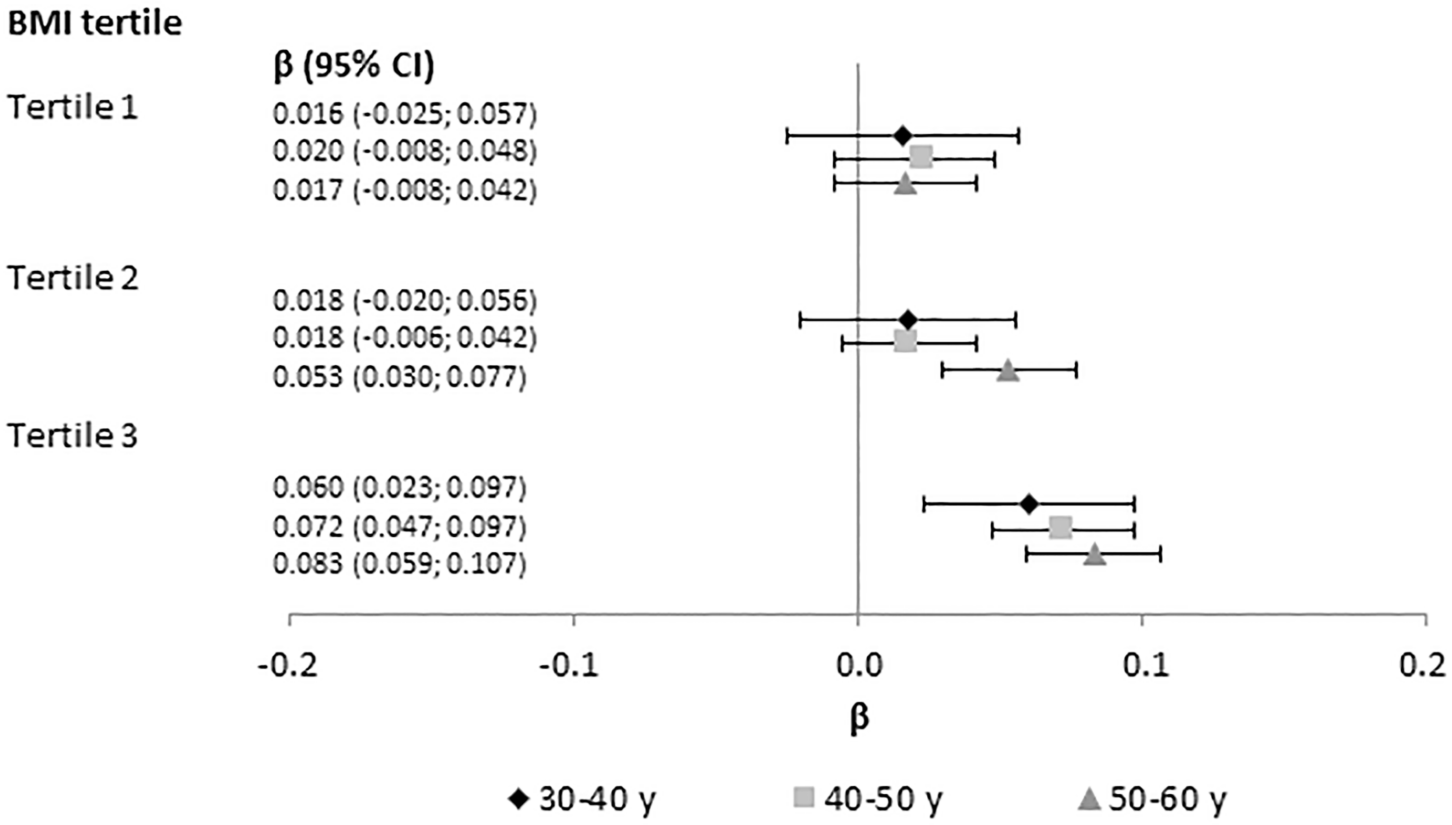
Beta (β) and 95% confidence intervals (CI) from linear regression with baseline plasma triglyceride level as exposure and plasma glucose change as outcome, by age (baseline- end of follow-up) and tertile of baseline BMI. All analyses were adjusted for baseline smoking status and baseline level of glucose, BMI, and cholesterol. Triglycerides, and annual glucose change as outcome, were log-transformed and entered into the model on their Z transformed scale, standardized by sex and cohort. Each analysis excluded individuals with values more extreme than ±3 standard deviations of the baseline level of triglycerides or glucose or of change in glucose level. The number of individuals in each tertile analysis was: 30–40 y, 2282–2376; 40–50 y, 4082–4217; 50–60 y, 4674–4893. The range of cohort- and sex-specific BMI tertile cut-points were for T1-2: 30 y, 20.6–23.3 kg/m^2^; 40 y, 21.8–24.2 kg/m^2^, 50 y, 23.2–24.8 kg/m^2^; and for T2-3; 30 y, 23.2–25.8 kg/m^2^, 40 y, 24.9–26.9 kg/m^2^, 50 y, 26.6–27.6 kg/m^2^. Abbreviations: BMI, body mass index; CI, confidence interval; y, years.

Interaction tests between baseline BMI and other metabolic factors showed many interactions with *P*-values below 0.05, which, however, should be evaluated in the light of the multiple tests performed (S2 Table). BMI and triglycerides interacted in relation to glucose changes in the full population in all age groups, and the interaction was evident in both cohorts and in both men and women, which altogether was considered evident of an interaction that was unlikely to be due to chance. The analysis of baseline triglycerides levels in relation to 10-year glucose changes by tertile of baseline BMI showed that higher triglyceride levels were significantly associated with increases in blood glucose, generally only among those in the third tertile of BMI (Fig 2).

## Discussion

This study is the largest European study on how metabolic factors predict changes in other metabolic factors. By use of 10-year follow-up data from almost 60,000 men and women grouped into three age groups we found that BMI was the strongest indicator of future changes in glucose and mid-blood pressure and, to a lesser extent, triglycerides levels. In addition, triglycerides were a strong indicator of glucose changes among individuals with elevated BMI, particularly in the older age groups.

In addition to the aforementioned studies [4–6], several other Asian and north American studies have explored longitudinal associations between metabolic factors and found BMI to be one of the strongest predictors for future changes in other metabolic factors [14–17]. For instance, after a follow-up period of six years, the Baltimore Longitudinal Study on Aging showed that predictors of developing metabolic syndrome were higher baseline abdominal obesity or triglycerides and lower HDL cholesterol. Men were more likely than women to have the metabolic syndrome without obesity, whereas women were more likely than men to have the metabolic syndrome without an altered glucose metabolism [17].

Our findings are in line with existing studies, as we also observed that BMI was the strongest indicator for changes in other metabolic factors—several of which are considered to be part of the metabolic syndrome (i.e. diabetes, high triglycerides, and hypertension). The inverse association with cholesterol may be explained by a reduction in HDL cholesterol [18]–which was not available in our data set. The concentration of HDL cholesterol is low in obese people and intra-abdominal visceral fat deposition is associated with low levels of HDL-cholesterol. The decreased HDL levels in obese persons have been attributed to an increased uptake of HDL2 by adipocytes and an increased catabolism of apolipoprotein A-I [18]. The positive association between triglycerides and future glucose changes in older subjects is also in line with existing observations—though to our knowledge no studies to date were able to evaluate longitudinal changes covering the age span of 30 to 60 years, and especially studies of the youngest age group are lacking. The Israeli Metabolic, Life-Style, and Nutritional Assessment in Young Adults (MELANY) study, found that alterations in triglyceride levels predicted risk for diabetes [19]. Most other studies published to date only provide cross-sectional findings of a link between triglycerides and glucose levels in obese subjects [20, 21].

Several experimental studies have also indicated a link between various metabolic factors. In particular BMI has been found to be strongly associated with risk of diabetes and insulin resistance. More specifically, obese individuals have higher levels of non-esterified fatty acids, glycerol, hormones, cytokines, and pro-inflammatory markers—which may contribute to the failure of β-islet cells in the pancreas and consequently the development of diabetes [22]. Pre-clinical evidence is also consistent for the development of obesity-induced hypertension [23]. Increased renal sodium reabsorption and impaired pressure natriuresis may play key roles in the link between hypertension and obesity [23].

The strongest indicator of future changes was found between age 30 and 40. These observations are also the most important ones as they were unlikely to be influenced by comorbidity and use of medication. More specifically, the association between BMI and mid-blood pressure was strongest in ages 30–40. Blood pressure at this age was the only metabolic factor that did not increase over time, suggesting that at an age where blood pressure remains stable, obesity affects future changes of other metabolic factors [24]. Nevertheless, lifestyle interventions are likely to also have an impact in older people regarding metabolic risk factors, as even among the elderly we observed positive associations between the different metabolic factors.

Even though the current study was conducted in an observational setting, it can provide information to further generate hypotheses about potential underlying biological mechanisms. For example, given the recent increase in evidence for an inflammatory impact of obesity [25], it can be postulated that the associations observed in this study may be driven by an immunological component as also hypertension, dyslipidemia [26] and diabetes [27] have also been linked with dysregulation of the immune system.

The current study is unique due to the use of two large population-based cohorts with fasting samples, allowing for sex-specific analyses based on three age groups. However, the external validity of our findings may be limited, especially in non-Caucasian populations. A strength of our study was that we investigated the full range of exposure levels for the different metabolic factors, rather than the often used dichotomized values based on cut-offs without strong evidence [28]. A potential limitation is that the VIP was an intervention study in which lifestyle advice, and if needed pharmaceutical treatment, was provided. This may potentially weaken some of the associations observed, however several long-term dietary and lifestyle interventions have shown that people start disregarding the advice already after 12 months [29, 30]. This again reflects the need for better and more effective lifestyle intervention as our findings clearly suggest that targeted lifestyle interventions can help reduce development of metabolic aberrations in future. Another limitation is that no information was available on abdominal obesity, body shape, or body fat proportion, which is more relevant to metabolic aberrations than is general obesity. Lastly, the lack of complete data on potentially important confounders, especially drug use and comorbidities, may have distorted our results, particularly in the older age groups where these specific confounders are more prevalent.

In conclusion, in this large European study, BMI was the strongest indicator of future changes in metabolic factors—especially in those aged 30 to 40 years. Our study supports that lifestyle interventions preventing metabolic aberrations should focus on avoiding weight increases.

## Supporting information

S1 Fig Partial correlation coefficients between metabolic factors by sex and cohort at ages A) 30 years, B) 40 years, and C) 50 years. Metabolic factors were log-transformed and entered into the model on their Z transformed scale, standardized by sex and cohort. All analyses were adjusted for baseline smoking status. Participants with a value more extreme than ±3 standard deviations of a metabolic factor in a paired correlation were excluded (maximum 2% of individuals for an analysis). Grey shading highlight r≥0.10, with a darker grey tone for every 0.10 stronger correlation coefficient. Bold numbers denote *P*-values<0.05. Abbreviations: M, men; W, women; VIP, Västerbotten Intervention Project; VHM&PP, Vorarlberg Health Monitoring and Prevention Programme.
(DOCX)

S2 Fig Beta from linear regression with baseline A) body mass index, B) mid-blood pressure, C) glucose, D) total cholesterol, and E) triglycerides as exposure, and change in a metabolic factor as outcome, by age (baseline-end of follow-up), sex, and cohort. Analyses were adjusted for baseline smoking status and baseline level of the outcome metabolic factor and body mass index (except in A). Analyses of cholesterol and triglycerides as exposures were additionally mutually adjusted for baseline level of the counterpart factor. All metabolic factors, and annual change of the outcome metabolic factor, were log-transformed and entered into the model on their Z transformed scale, standardized by sex and cohort. Grey shadings highlight coefficients ≥0.05, with a darker grey tone for every 0.05 stronger association. Striped shading indicate inverse associations for coefficients ≤-0.05. Bold numbers denote *P*-values<0.05. Each analysis excluded individuals with values more extreme than ±3 standard deviations of the exposure, outcome, or baseline level of the outcome metabolic factor. The number of individuals in each analysis was: 30–40 years, VIP-M = 580–1294, VIP-W = 814–1297, VHM&PP-M = 1618–2572, VHM&PP-W = 1759–3244; 40–50 years, VIP-M = 2852–5285, VIP-W = 3822–5724, VHM&PP-M = 1657–2660, VHM&PP-W = 2538–3485; 50–60 years, VIP-M = 2997–5085, VIP-W = 4295–5618, VHM&PP-M = 1680–2784, VHM&PP-W = 2915–3596. Abbreviations: M, men; W, women; VIP, Västerbotten Intervention Project; VHM&PP, Vorarlberg Health Monitoring and Prevention Programme.
(DOCX)

S3 Fig Sensitivity analyses in the Västerbotten Intervention Project of associations in S2 Fig, with additional exclusions of participants on antihypertensive drugs at baseline in analyses of blood pressure as exposure, and of participants on antihypertensive drugs at baseline or at follow-up in analyses of blood pressure change as outcome, and with the corresponding exclusions of individuals with diabetes in analyses of glucose. The figure shows betas from linear regression with baseline A) body mass index, B) mid-blood pressure, C) glucose, D) total cholesterol, and E) triglycerides as exposure, and change in a metabolic factor as outcome, by age (baseline-end of follow-up) and sex for analyses where blood pressure or glucose was the exposure, or their change was the outcome. See S2 Fig for further information. The number of individuals were: 30–40 years-M = 557–1294, 30–40 years-W = 784–1296, 40–50 years-M = 2779–5279, 40–50 years-W = 3771–5720, 50–60 years-M = 3041–5046, 50–60 years-W = 3967–5605. Abbreviations: M, men; W, women.
(DOCX)

S1 Table Age- and sex-specific formulas used in the Västerbotten Intervention Project to convert blood pressure levels measured on Sept 1, 2009 onwards in sitting position, to levels measured before that date in supine position. (DOCX)

S2 Table Interactions with a *P-*value below 0.05 between body mass index and another metabolic factor at baseline, ordered by the number of times interacting i) in the full population and ii) within a cohort and sex. (DOCX)
