# Supplementary material for: Longitudinal study of body mass index, dyslipidemia, hyperglycemia, and hypertension in 60,000 men and women in Sweden and Austria
Source: PLoS One. 2018 Jun 13;13(6):e0197830. doi: 10.1371/journal.pone.0197830 (PMC5999071; doi:10.1371/journal.pone.0197830)
Supplement: S1 Fig — Metabolic factors were log-transformed and entered into the model on their Z transformed scale, standardized by sex and cohort. All analyses were adjusted for baseline smoking status. Participants with a value more extreme than ±3 standard deviations of a metabolic factor in a paired correlation were excluded (maximum 2% of individuals for an analysis). Grey shading highlight r≥0.10, with a darker grey tone for every 0.10 stronger correlation coefficient. Bold numbers denote P-values<0.05. Abbreviations: M, men; W, women; VIP, Västerbotten Intervention Project; VHM&PP, Vorarlberg Health Monitoring and Prevention Programme. (DOCX) [file pone.0197830.s001.docx]

S1A-C Fig. Partial correlation coefficients between metabolic factors by sex and cohort at ages A) 30 years, B) 40 years, and C) 50 years. Metabolic factors were log-transformed and entered into the model on their Z transformed scale, standardized by sex and cohort. All analyses were adjusted for baseline smoking status. Participants with a value more extreme than ±3 standard deviations of a metabolic factor in a paired correlation were excluded (maximum 2% of individuals for an analysis). Grey shading highlight r≥0.10, with a darker grey tone for every 0.10 stronger correlation coefficient. Bold numbers denote *P*-values<0.05. Abbreviations: M, men; W, women; VIP, Västerbotten Intervention Project; VHM&PP, Vorarlberg Health Monitoring and Prevention Programme.

**A) 30-years.** *N* VIP-M=1194-1642, *N* VIP W=1653-1797, *N* VHM&PP-M=3465-3502, *N* VHM&PP-W=4379-4448.

| Metabolic factor | Cohort | Mid-blood pressure | | Glucose | | Cholesterol | | Triglycerides | |
| --- | --- | --- | --- | --- | --- | --- | --- | --- | --- |
|  |  | M | W | M | W | M | W | M | W |
| Body mass index | VIP | **0.26** | **0.28** | **0.06** | **0.13** | **0.23** | **0.14** | **0.41** | **0.27** |
|  | VHM&PP | **0.28** | **0.26** | **0.06** | **0.09** | **0.22** | **0.13** | **0.33** | **0.24** |
| Mid-blood pressure | VIP |  |  | 0.04 | **0.09** | **0.10** | **0.12** | **0.14** | **0.12** |
|  | VHM&PP |  |  | **0.08** | **0.06** | **0.12** | **0.08** | **0.15** | **0.12** |
| Glucose | VIP |  |  |  |  | -0.01 | **-0.06** | **0.09** | **0.07** |
|  | VHM&PP |  |  |  |  | 0.02 | 0.00 | 0.01 | 0.02 |
| Cholesterol | VIP |  |  |  |  |  |  | **0.39** | **0.24** |
|  | VHM&PP |  |  |  |  |  |  | **0.41** | **0.37** |

**B) 40-years.** *N* VIP-M=7172-8301, *N* VIP-W=8982-9398, *N* VHM&PP-M=7204-7273, *N* VHM&PP-W=9073-9144.

| Metabolic factor | Cohort | Mid-blood pressure | | Glucose | | Cholesterol | | Triglycerides | |
| --- | --- | --- | --- | --- | --- | --- | --- | --- | --- |
|  |  | M | W | M | W | M | W | M | W |
| Body mass index | VIP | **0.30** | **0.29** | **0.14** | **0.17** | **0.14** | **0.14** | **0.37** | **0.35** |
|  | VHM&PP | **0.30** | **0.34** | **0.15** | **0.16** | **0.16** | **0.11** | **0.35** | **0.33** |
| Mid-blood pressure | VIP |  |  | **0.06** | **0.07** | **0.13** | **0.13** | **0.19** | **0.17** |
|  | VHM&PP |  |  | **0.08** | **0.08** | **0.14** | **0.09** | **0.19** | **0.18** |
| Glucose | VIP |  |  |  |  | -0.02 | -0.01 | **0.10** | **0.13** |
|  | VHM&PP |  |  |  |  | 0.01 | 0.01 | **0.06** | **0.06** |
| Cholesterol | VIP |  |  |  |  |  |  | **0.31** | **0.26** |
|  | VHM&PP |  |  |  |  |  |  | **0.39** | **0.34** |

**C) 50-years.** *N* VIP-M=13,642-14,759, *N* VIP-W=15,919-16,416, *N* VHM&PP-M=7608-7708, *N* VHM&PP-W=9802-9907.

| Metabolic factor | Cohort | Mid-blood pressure | | Glucose | | Cholesterol | | Triglycerides | |
| --- | --- | --- | --- | --- | --- | --- | --- | --- | --- |
|  |  | M | W | M | W | M | W | M | W |
| Body mass index | VIP | **0.31** | **0.29** | **0.14** | **0.20** | **0.04** | **0.08** | **0.35** | **0.35** |
|  | VHM&PP | **0.31** | **0.34** | **0.18** | **0.19** | **0.07** | **0.08** | **0.31** | **0.34** |
| Mid-blood pressure | VIP |  |  | **0.09** | **0.12** | **0.10** | **0.11** | **0.19** | **0.20** |
|  | VHM&PP |  |  | **0.11** | **0.09** | **0.11** | **0.09** | **0.19** | **0.22** |
| Glucose | VIP |  |  |  |  | -0.08 | -0.05 | **0.04** | **0.12** |
|  | VHM&PP |  |  |  |  | 0.01 | 0.01 | **0.09** | **0.10** |
| Cholesterol | VIP |  |  |  |  |  |  | **0.30** | **0.27** |
|  | VHM&PP |  |  |  |  |  |  | **0.36** | **0.35** |
