# Supplementary material for: Longitudinal study of body mass index, dyslipidemia, hyperglycemia, and hypertension in 60,000 men and women in Sweden and Austria
Source: PLoS One. 2018 Jun 13;13(6):e0197830. doi: 10.1371/journal.pone.0197830 (PMC5999071; doi:10.1371/journal.pone.0197830)
Supplement: S2 Fig — Analyses were adjusted for baseline smoking status and baseline level of the outcome metabolic factor and body mass index (except in A). Analyses of cholesterol and triglycerides as exposures were additionally mutually adjusted for baseline level of the counterpart factor. All metabolic factors, and annual change of the outcome metabolic factor, were log-transformed and entered into the model on their Z transformed scale, standardized by sex and cohort. Grey shadings highlight coefficients ≥0.05, with a darker grey tone for every 0.05 stronger association. Striped shading indicate inverse associations for coefficients ≤-0.05. Bold numbers denote P-values<0.05. Each analysis excluded individuals with values more extreme than ±3 standard deviations of the exposure, outcome, or baseline level of the outcome metabolic factor. The number of individuals in each analysis was: 30–40 years, VIP-M = 580–1294, VIP-W = 814–1297, VHM&PP-M = 1618–2572, VHM&PP-W = 1759–3244; 40–50 years, VIP-M = 2852–5285, VIP-W = 3822–5724, VHM&PP-M = 1657–2660, VHM&PP-W = 2538–3485; 50–60 years, VIP-M = 2997–5085, VIP-W = 4295–5618, VHM&PP-M = 1680–2784, VHM&PP-W = 2915–3596. Abbreviations: M, men; W, women; VIP, Västerbotten Intervention Project; VHM&PP, Vorarlberg Health Monitoring and Prevention Programme. (DOCX) [file pone.0197830.s002.docx]

S2A-E Fig. Beta from linear regression with baseline A) body mass index, B) mid-blood pressure, C) glucose, D) total cholesterol, and E) triglycerides as exposure, and change in a metabolic factor as outcome, by age (baseline-end of follow-up), sex, and cohort. Analyses were adjusted for baseline smoking status and baseline level of the outcome metabolic factor and body mass index (except in A). Analyses of cholesterol and triglycerides as exposures were additionally mutually adjusted for baseline level of the counterpart factor. All metabolic factors, and annual change of the outcome metabolic factor, were log-transformed and entered into the model on their Z transformed scale, standardized by sex and cohort. Grey shadings highlight coefficients ≥0.05, with a darker grey tone for every 0.05 stronger association. Striped shading indicate inverse associations for coefficients ≤-0.05. Bold numbers denote *P*-values<0.05. Each analysis excluded individuals with values more extreme than ±3 standard deviations of the exposure, outcome, or baseline level of the outcome metabolic factor. The number of individuals in each analysis was: 30-40 years, VIP-M=580-1294, VIP-W=814-1297, VHM&PP-M=1618-2572, VHM&PP-W=1759-3244; 40-50 years, VIP-M=2852-5285, VIP-W=3822-5724, VHM&PP-M=1657-2660, VHM&PP-W=2538-3485; 50-60 years, VIP-M=2997-5085, VIP-W=4295-5618, VHM&PP-M=1680-2784, VHM&PP-W=2915-3596. Abbreviations: M, men; W, women; VIP, Västerbotten Intervention Project; VHM&PP, Vorarlberg Health Monitoring and Prevention Programme.

A) Body mass index

| Outcome factor | Cohort | 30-40 years | | 40-50 years | | 50-60 years | |
| --- | --- | --- | --- | --- | --- | --- | --- |
|  |  | M | W | M | W | M | W |
| Mid-blood pressure | VIP | **0.16** | **0.14** | **0.12** | **0.11** | **0.06** | **0.06** |
|  | VHM&PP | **0.13** | **0.20** | **0.15** | **0.13** | **0.15** | **0.13** |
| Glucose | VIP | **0.03** | **0.02** | **0.06** | **0.07** | **0.09** | **0.06** |
|  | VHM&PP | **0.07** | **0.13** | **0.16** | **0.18** | **0.22** | **0.19** |
| Cholesterol | VIP | 0.00 | 0.02 | **-0.04** | 0.00 | **-0.04** | **-0.05** |
|  | VHM&PP | **-0.08** | 0.04 | **-0.07** | **-0.04** | -0.02 | **-0.06** |
| Triglycerides | VIP | 0.01 | **0.14** | 0.03 | **0.04** | **0.05** | **0.03** |
|  | VHM&PP | **0.09** | **0.07** | 0.03 | **0.07** | 0.02 | 0.04 |

B) Mid-blood pressure

| Outcome factor | Cohort | 30-40 years | | 40-50 years | | 50-60 years | |
| --- | --- | --- | --- | --- | --- | --- | --- |
|  |  | M | W | M | W | M | W |
| Weight | VIP | -0.03 | -0.02 | 0.00 | 0.00 | 0.01 | 0.02 |
|  | VHM&PP | 0.01 | 0.00 | -0.01 | 0.01 | 0.02 | 0.02 |
| Glucose | VIP | 0.00 | **0.01** | **0.03** | **0.02** | **0.04** | **0.04** |
|  | VHM&PP | -0.01 | 0.00 | **0.06** | **0.04** | **0.07** | **0.05** |
| Cholesterol | VIP | 0.00 | -0.01 | 0.00 | 0.02 | -0.02 | **0.03** |
|  | VHM&PP | -0.02 | 0.00 | 0.02 | 0.02 | 0.03 | -0.01 |
| Triglycerides | VIP | 0.02 | 0.03 | 0.00 | **0.01** | 0.01 | **0.03** |
|  | VHM&PP | 0.04 | -0.01 | 0.04 | 0.00 | 0.04 | 0.02 |

C) Glucose

| Outcome factor | Cohort | 30-40 years | | 40-50 years | | 50-60 years | |
| --- | --- | --- | --- | --- | --- | --- | --- |
|  |  | M | W | M | W | M | W |
| Weight | VIP | -0.01 | 0.04 | 0.02 | **0.03** | 0.01 | -0.02 |
|  | VHM&PP | 0.02 | 0.02 | -0.01 | 0.01 | -0.01 | 0.00 |
| Mid-blood pressure | VIP | 0.04 | 0.01 | 0.00 | 0.03 | 0.01 | 0.00 |
|  | VHM&PP | **-0.05** | **0.07** | -0.02 | 0.03 | **0.06** | 0.03 |
| Cholesterol | VIP | -0.02 | -0.02 | 0.02 | **0.04** | 0.02 | **0.03** |
|  | VHM&PP | 0.04 | 0.01 | -0.01 | **0.05** | -0.01 | 0.00 |
| Triglycerides | VIP | -0.02 | -0.01 | -0.01 | 0.00 | 0.00 | 0.01 |
|  | VHM&PP | 0.01 | -0.02 | 0.00 | 0.01 | 0.02 | **0.07** |

D) Cholesterol

| Outcome factor | Cohort | 30-40 years | | 40-50 years | | 50-60 years | |
| --- | --- | --- | --- | --- | --- | --- | --- |
|  |  | M | W | M | W | M | W |
| Weight | VIP | -0.05 | -0.01 | -0.02 | **-0.04** | -0.02 | -0.01 |
|  | VHM&PP | **-0.04** | -0.03 | -0.03 | -0.01 | **-0.05** | -0.02 |
| Mid-blood pressure | VIP | 0.00 | -0.01 | 0.01 | 0.01 | 0.00 | -0.01 |
|  | VHM&PP | 0.01 | 0.00 | 0.01 | -0.02 | 0.00 | -0.02 |
| Glucose | VIP | 0.01 | **-0.02** | -0.02 | **-0.02** | -0.01 | **-0.02** |
|  | VHM&PP | -0.02 | 0.01 | 0.02 | **-0.04** | -0.03 | 0.01 |
| Triglycerides | VIP | 0.01 | **0.10** | **0.03** | **0.02** | **0.04** | **0.02** |
|  | VHM&PP | 0.05 | 0.01 | 0.01 | 0.04 | 0.01 | -0.01 |

E) Triglycerides

| Outcome factor | Cohort | 30-40 years | | 40-50 years | | 50-60 years | |
| --- | --- | --- | --- | --- | --- | --- | --- |
|  |  | M | W | M | W | M | W |
| Weight | VIP | -0.01 | 0.00 | -0.02 | -0.02 | 0.01 | -0.01 |
|  | VHM&PP | 0.02 | 0.02 | -0.02 | -0.02 | 0.01 | 0.00 |
| Mid-blood pressure | VIP | 0.01 | **0.05** | **0.04** | 0.03 | 0.02 | **0.06** |
|  | VHM&PP | 0.03 | 0.01 | 0.04 | **0.05** | **0.06** | **0.08** |
| Glucose | VIP | 0.01 | **0.03** | **0.03** | **0.03** | **0.03** | **0.03** |
|  | VHM&PP | 0.03 | 0.02 | **0.09** | **0.06** | **0.09** | **0.13** |
| Cholesterol | VIP | **0.07** | 0.02 | **0.07** | **0.06** | **0.04** | **0.04** |
|  | VHM&PP | 0.03 | 0.04 | 0.03 | **0.07** | 0.04 | **0.05** |

A) Body mass index

| Outcome factor | 30-40 years | | 40-50 years | | 50-60 years | |
| --- | --- | --- | --- | --- | --- | --- |
|  | M | W | M | W | M | W |
| Mid-blood pressure | **0.16** | **0.14** | **0.13** | **0.12** | **0.08** | **0.06** |
| Glucose | **0.02** | **0.02** | **0.04** | **0.06** | **0.07** | **0.04** |

B) Mid-blood pressure

| Outcome factor | 30-40 years | | 40-50 years | | 50-60 years | |
| --- | --- | --- | --- | --- | --- | --- |
|  | M | W | M | W | M | W |
| Weight | -0.03 | -0.02 | 0.00 | 0.00 | 0.00 | 0.03 |
| Glucose | 0.01 | 0.01 | **0.02** | **0.02** | **0.04** | **0.03** |
| Cholesterol | 0.00 | -0.01 | 0.00 | 0.02 | -0.03 | **0.03** |
| Triglycerides | 0.02 | 0.03 | 0.00 | **0.01** | 0.00 | **0.03** |

C) Glucose

| Outcome factor | 30-40 years | | 40-50 years | | 50-60 years | |
| --- | --- | --- | --- | --- | --- | --- |
|  | M | W | M | W | M | W |
| Weight | -0.01 | 0.04 | 0.02 | 0.03 | 0.01 | -0.02 |
| Mid-blood pressure | 0.04 | 0.01 | 0.00 | **0.03** | 0.01 | 0.02 |
| Cholesterol | -0.02 | -0.02 | 0.02 | **0.04** | 0.02 | **0.03** |
| Triglycerides | -0.02 | -0.01 | -0.01 | 0.00 | 0.00 | 0.01 |

D) Cholesterol

| Outcome factor | 30-40 years | | 40-50 years | | 50-60 years | |
| --- | --- | --- | --- | --- | --- | --- |
|  | M | W | M | W | M | W |
| Mid-blood pressure | -0.01 | -0.01 | 0.00 | 0.00 | 0.01 | -0.01 |
| Glucose | 0.01 | -0.01 | -0.01 | **-0.02** | -0.01 | **-0.02** |

E) Triglycerides

| Outcome factor | 30-40 years | | 40-50 years | | 50-60 years | |
| --- | --- | --- | --- | --- | --- | --- |
|  | M | W | M | W | M | W |
| Mid-blood pressure | -0.02 | 0.05 | **0.04** | 0.03 | 0.01 | **0.06** |
| Glucose | 0.01 | **0.02** | **0.02** | **0.03** | **0.03** | **0.02** |

S3A-E Fig. Sensitivity analyses in the Västerbotten Intervention Project of associations in Figure S2, with additional exclusions of participants on antihypertensive drugs at baseline in analyses of blood pressure as exposure, and of participants on antihypertensive drugs at baseline or at follow-up in analyses of blood pressure change as outcome, and with the corresponding exclusions of individuals with diabetes in analyses of glucose. The figure shows betas from linear regression with baseline A) body mass index, B) mid-blood pressure, C) glucose, D) total cholesterol, and E) triglycerides as exposure, and change in a metabolic factor as outcome, by age (baseline-end of follow-up) and sex for analyses where blood pressure or glucose was the exposure, or their change was the outcome. See Figure S2 for further information. The number of individuals were: 30-40 years-M=557-1294, 30-40 years-W=784-1296, 40-50 years-M=2779-5279, 40-50 years-W=3771-5720, 50-60 years-M=3041-5046, 50-60 years-W=3967-5605. Abbreviations: M, men; W, women.
