# Supplementary material for: Longitudinal study of body mass index, dyslipidemia, hyperglycemia, and hypertension in 60,000 men and women in Sweden and Austria
Source: PLoS One. 2018 Jun 13;13(6):e0197830. doi: 10.1371/journal.pone.0197830 (PMC5999071; doi:10.1371/journal.pone.0197830)
Supplement: S3 Fig — The figure shows betas from linear regression with baseline A) body mass index, B) mid-blood pressure, C) glucose, D) total cholesterol, and E) triglycerides as exposure, and change in a metabolic factor as outcome, by age (baseline-end of follow-up) and sex for analyses where blood pressure or glucose was the exposure, or their change was the outcome. See S2 Fig for further information. The number of individuals were: 30–40 years-M = 557–1294, 30–40 years-W = 784–1296, 40–50 years-M = 2779–5279, 40–50 years-W = 3771–5720, 50–60 years-M = 3041–5046, 50–60 years-W = 3967–5605. Abbreviations: M, men; W, women. (DOCX) [file pone.0197830.s003.docx]

S3A-E Fig. Sensitivity analyses in the Västerbotten Intervention Project of associations in Figure S2, with additional exclusions of participants on antihypertensive drugs at baseline in analyses of blood pressure as exposure, and of participants on antihypertensive drugs at baseline or at follow-up in analyses of blood pressure change as outcome, and with the corresponding exclusions of individuals with diabetes in analyses of glucose. The figure shows betas from linear regression with baseline A) body mass index, B) mid-blood pressure, C) glucose, D) total cholesterol, and E) triglycerides as exposure, and change in a metabolic factor as outcome, by age (baseline-end of follow-up) and sex for analyses where blood pressure or glucose was the exposure, or their change was the outcome. See Figure S2 for further information. The number of individuals were: 30-40 years-M=557-1294, 30-40 years-W=784-1296, 40-50 years-M=2779-5279, 40-50 years-W=3771-5720, 50-60 years-M=3041-5046, 50-60 years-W=3967-5605. Abbreviations: M, men; W, women.

A) Body mass index

| Outcome factor | 30-40 years | | 40-50 years | | 50-60 years | |
| --- | --- | --- | --- | --- | --- | --- |
|  | M | W | M | W | M | W |
| Mid-blood pressure | **0.16** | **0.14** | **0.13** | **0.12** | **0.08** | **0.06** |
| Glucose | **0.02** | **0.02** | **0.04** | **0.06** | **0.07** | **0.04** |

B) Mid-blood pressure

| Outcome factor | 30-40 years | | 40-50 years | | 50-60 years | |
| --- | --- | --- | --- | --- | --- | --- |
|  | M | W | M | W | M | W |
| Weight | -0.03 | -0.02 | 0.00 | 0.00 | 0.00 | 0.03 |
| Glucose | 0.01 | 0.01 | **0.02** | **0.02** | **0.04** | **0.03** |
| Cholesterol | 0.00 | -0.01 | 0.00 | 0.02 | -0.03 | **0.03** |
| Triglycerides | 0.02 | 0.03 | 0.00 | **0.01** | 0.00 | **0.03** |

C) Glucose

| Outcome factor | 30-40 years | | 40-50 years | | 50-60 years | |
| --- | --- | --- | --- | --- | --- | --- |
|  | M | W | M | W | M | W |
| Weight | -0.01 | 0.04 | 0.02 | 0.03 | 0.01 | -0.02 |
| Mid-blood pressure | 0.04 | 0.01 | 0.00 | **0.03** | 0.01 | 0.02 |
| Cholesterol | -0.02 | -0.02 | 0.02 | **0.04** | 0.02 | **0.03** |
| Triglycerides | -0.02 | -0.01 | -0.01 | 0.00 | 0.00 | 0.01 |

D) Cholesterol

| Outcome factor | 30-40 years | | 40-50 years | | 50-60 years | |
| --- | --- | --- | --- | --- | --- | --- |
|  | M | W | M | W | M | W |
| Mid-blood pressure | -0.01 | -0.01 | 0.00 | 0.00 | 0.01 | -0.01 |
| Glucose | 0.01 | -0.01 | -0.01 | **-0.02** | -0.01 | **-0.02** |

E) Triglycerides

| Outcome factor | 30-40 years | | 40-50 years | | 50-60 years | |
| --- | --- | --- | --- | --- | --- | --- |
|  | M | W | M | W | M | W |
| Mid-blood pressure | -0.02 | 0.05 | **0.04** | 0.03 | 0.01 | **0.06** |
| Glucose | 0.01 | **0.02** | **0.02** | **0.03** | **0.03** | **0.02** |
