# Supplementary material for: Longitudinal study of body mass index, dyslipidemia, hyperglycemia, and hypertension in 60,000 men and women in Sweden and Austria
Source: PLoS One. 2018 Jun 13;13(6):e0197830. doi: 10.1371/journal.pone.0197830 (PMC5999071; doi:10.1371/journal.pone.0197830)
Supplement: S1 Table — (DOCX) [file pone.0197830.s004.docx]

S1 Table. Age- and sex-specific formulas used in the Västerbotten Intervention Project to convert blood pressure levels measured on Sept 1, 2009 onwards in sitting position, to levels measured before that date in supine position

| **Age** | **Sex** | **Conversion formula** |
| --- | --- | --- |
| 30-40 years | Men | 24.595 + (0.792 × systolic blood pressure level)  17.282 + (0.753 × diastolic blood pressure level) |
|  | Women | 8.669 + (0.919 × systolic blood pressure level) 5.784 + (0.890 × diastolic blood pressure level) |
| 50 years | Men | 9.850 + (0.910 × systolic blood pressure level) 12.363 + (0.812 × diastolic blood pressure level) |
|  | Women | 16.051 + (0.859 × systolic blood pressure level) 13.566 + (0.798 × diastolic blood pressure level) |
| 60 years | Men | 7.763 + (0.936 × systolic blood pressure level) 9.029 + (0.864 × diastolic blood pressure level) |
|  | Women | 9.999 + (0.914 × systolic blood pressure level) 7.992 + (0.870 × diastolic blood pressure level) |
