# Supplementary material for: Longitudinal study of body mass index, dyslipidemia, hyperglycemia, and hypertension in 60,000 men and women in Sweden and Austria
Source: PLoS One. 2018 Jun 13;13(6):e0197830. doi: 10.1371/journal.pone.0197830 (PMC5999071; doi:10.1371/journal.pone.0197830)
Supplement: S2 Table — (DOCX) [file pone.0197830.s005.docx]

S2 Table. Interactions with a *P-*value below 0.05 between body mass index and another metabolic factor at baseline, ordered by the number of times interacting i) in the full population and ii) within a cohort and sex

| **Interacting factor - outcome** | **Baseline age** | **Cohort/-s** | **Sex** | ***P*-value** |
| --- | --- | --- | --- | --- |
| Triglycerides - glucose change | 30 | Both | Both | 0.007 |
|  | 30 | VIP | Women | 0.03 |
|  | 30 | VHM&PP | Women | <0.001 |
|  | 40 | Both | Both | <0.001 |
|  | 40 | VIP | Men | 0.001 |
|  | 40 | VIP | Women | 0.01 |
|  | 40 | VHM&PP | Women | 0.002 |
|  | 50 | Both | Both | <0.001 |
|  | 50 | VIP | Men | 0.002 |
|  | 50 | VIP | Women | 0.001 |
|  | 50 | VHM&PP | Men | 0.04 |
|  | 50 | VHM&PP | Women | <0.001 |
| Mid-blood pressure - glucose change | 40 | Both | Both | 0.003 |
|  | 40 | VIP | Men | 0.001 |
|  | 40 | VIP | Women | 0.04 |
|  | 50 | Both | Both | <0.001 |
|  | 50 | VIP | Men | 0.046 |
|  | 50 | VIP | Women | <0.001 |
|  | 50 | VHM&PP | Men | 0.001 |
| Mid-blood pressure – weight change | 30 | Both | Both | 0.02 |
|  | 30 | VIP | Men | <0.001 |
|  | 50 | VIP | Men | 0.04 |
|  | 50 | VIP | Women | <0.001 |
| Cholesterol – triglyceride change | 40 | Both | Both | 0.048 |
|  | 40 | VIP | Men | 0.005 |
| Glucose – mid-blood pressure change | 50 | Both | Both | 0.01 |
| Triglycerides – weight change | 40 | VIP | Women | <0.001 |
|  | 40 | VHM&PP | Women | 0.009 |
|  | 50 | VIP | Women | 0.03 |
| Glucose – cholesterol change | 30 | VIP | Men | 0.04 |
|  | 30 | VHM&PP | Women | 0.005 |
| Glucose – weight change | 40 | VIP | Women | 0.004 |
|  | 50 | VHM&PP | Women | 0.045 |
| Cholesterol – weight change | 40 | VIP | Women | 0.03 |
|  | 50 | VHM&PP | Women | 0.007 |
| Glucose – triglyceride change | 30 | VHM&PP | Women | 0.02 |
| Cholesterol – glucose change | 30 | VHM&PP | Women | 0.02 |

Abbreviations: VIP, Västerbotten Intervention Project; VHM&PP, Vorarlberg Health Monitoring and Prevention Programme
